# Biodegradation of Polymers Used in Oil and Gas Operations: Towards Enzyme Biotechnology Development and Field Application

**DOI:** 10.3390/polym14091871

**Published:** 2022-05-03

**Authors:** Carolina Berdugo-Clavijo, Gabrielle Scheffer, Arindom Sen, Lisa M. Gieg

**Affiliations:** 1 Department of Biological Sciences, University of Calgary, 2500 University Drive NW, Calgary, AB T2N 1N4, Canada; caro1208@gmail.com (C.B.-C.); gabrielle.scheffe1@ucalgary.ca (G.S.); 2 Department of Chemical and Petroleum Engineering, University of Calgary, 2500 University Drive NW, Calgary, AB T2N 1N4, Canada; asen@ucalgary.ca

**Keywords:** polymer, biodegradation, enzyme biotechnology, polyacrylamide (PAM), carboxymethyl cellulose (CMC), guar, hydraulic fracturing, filter cake breakers

## Abstract

Linear and crosslinked polymers are commonly used in the oil and gas industry. Guar-derived polymers have been extensively utilized in hydraulic fracturing processes, and recently polyacrylamide and cellulose-based polymers have also found utility. As these polymers are used during various phases of the hydraulic fracturing process, they can accumulate at formation fracture faces, resulting in undesired filter cakes that impede oil and gas recovery. Although acids and chemical oxidizers are often added in the fracturing fluids to degrade or ‘break’ polymer filter cakes, the constant use of these chemicals can be hazardous and can result in formation damage and corrosion of infrastructure. Alternately, the use of enzymes is an attractive and environmentally friendly technology that can be used to treat polymer accumulations. While guar-linkage-specific enzyme breakers isolated from bacteria have been shown to successfully cleave guar-based polymers and decrease their molecular weight and viscosity at reservoir conditions, new enzymes that target a broader range of polymers currently used in hydraulic fracturing operations still require research and development for effective application. This review article describes the current state-of-knowledge on the mechanisms and enzymes involved in biodegradation of guar gum, polyacrylamide (and hydrolyzed polyacrylamide), and carboxymethyl cellulose polymers. In addition, advantages and challenges in the development and application of enzyme breaker technologies are discussed.

## 1. Introduction

Hydraulic fracturing (fracking) is a hydrocarbon extraction technique used to recover crude oil and/or gas from tight subsurface formations [1]. This energy recovery technology consists of injecting a water-based fluid under high pressure to create cracks, or fractures, in the reservoir rock [2]. Once the rock is fractured, the fluid penetrates the cracks, creating larger fractures away from the wellbore. The formed fractures increase the surface area and hydraulic conductivity within the reservoir, allowing for improved oil and gas mobility and recovery [3]. Polymers with gelling agent properties and linear or crosslinking structures are used in different steps of the hydraulic fracturing process (Table 1). The earliest step at which polymers are involved is during the drilling of a well, wherein polymers are added to the drilling muds as lubricants to facilitate operation [4,5]. In some cases, polymers are also used to help with the packing of the sand (gravel packing) and avoid accumulation of formation sand that can cause damage in the well [6]. Polymers are commonly added to fracturing fluids as proppant delivery agents to increase fluid viscosity and to help the proppant reach the fractures properly. Friction-reducing polymers such as polyacrylamide (PAM), or other acrylamide-based polymers, are added to the well to enhance the flow of the fluids through the pipes or tubing [7]. Thickeners and gelling agents facilitate proppant delivery as well, but are mainly added to the fluids to reduce leakoff, a process defined as the absorption of fluid into the rock matrix [8]. Cellulose-based polymers, guar gum, and PAM are all used as thickeners and gelling agents in hydraulic fracturing operations [8]. Fluid-loss additives help to reduce the loss of fluids when fracturing occurs in porous formations; thus, when thickeners are not sufficient to prevent leakoff, these types of additives are added [8]. Polymers are also used as foaming agents when fluid loss can be problematic or where water is scarce, as foamed fluids require less water than conventional water-based fracturing fluids [8].

As polymers are used in different steps of hydraulic fracturing (Table 1), they can accumulate in the reservoir and negatively impact oil and gas production. Undesired polymers can accumulate by leakoff, which happens when water from the fracturing fluid is absorbed into a porous matrix, resulting in sheared polymer molecules that accumulate in the rock pores. Thus, when polymers accumulate on the rock matrix, oil and gas recovery is no longer possible [9]. The polymers and gelling agents that are added in the fracking fluids help to prevent leakoff of the fluids into the surrounding rock by forming a layer of filter cake that keeps the fluid in place [3]. However, sometimes these filter cakes are not effective, and the fracking fluids can still be absorbed in the rock. Absorption of the fluids containing polymers can result in internal undesired filter cakes which can accumulate within the fractures [3]. Acids and oxidizers such as hydrogen chloride and ammonium persulfate are typically applied within the fracking fluids, and activated with catalyzers (such as amine, acetoacetate, or iron) to break the residual polymers in the reservoirs. These oxidative breakers yield free-radical compounds that can split carbon-carbon bonds in the polymers and degrade the filter cakes [10]. However, oxidizers are not specific polymer breakers, they must be added at very high concentrations, and can result in formation of undesired products that can lead to corrosion and lower conductivity problems, and can also be dangerous to the environment and hazardous to workers [11]. An attractive, more ecologically friendly approach for the treatment of residual polymers is through the use of microbial enzymes that can selectively degrade the polymer backbone to decrease its molecular weight and viscosity, resulting in filter cake removal in situ.

Enzymes are proteins found in all living organisms that act as catalysts to accelerate chemical reactions from 10^8^ to 10^20^ times higher than that which would spontaneously occur [12]. By forming a substrate–enzyme complex, enzymes lower a reactant’s activation energy, accelerating reactions [12]. As microorganisms can thrive under a variety of environmental conditions characteristic of subsurface petroliferous reservoirs, such as at low redox potentials, and across high salinity and temperature ranges, enzymes secreted by microorganisms have great potential as polymer breakers in such environments. Since the early 1990s, the use of enzyme breakers has been proposed for degrading residual polymers in hydraulic fracturing operations. Most notably, guar-linkage-specific enzymes (GLS) were initially obtained and used for the cleavage of specific guar linkages resulting in significant molecular weight reduction in guar-based filter cakes [10]. The successful degradation of guar-based polymers by GLS was shown in laboratory and oil reservoir case studies across the United States [13]. As other polymers such as PAM and cellulose-based polymers are now also being used in oil and gas recovery processes, there is high industry interest for developing new enzyme breakers to treat these polymers. In addition, with new advances in genetics and biochemistry, enzymes involved in polymer degradation can be optimized to be more stable at specific oil and gas reservoir conditions (e.g., remain stable at the higher temperatures, pressures, and/or salinities that characterize most petroleum reservoirs) and to produce higher yields for field application to prevent filter cakes and reservoir blockage.

**Figure 1:**
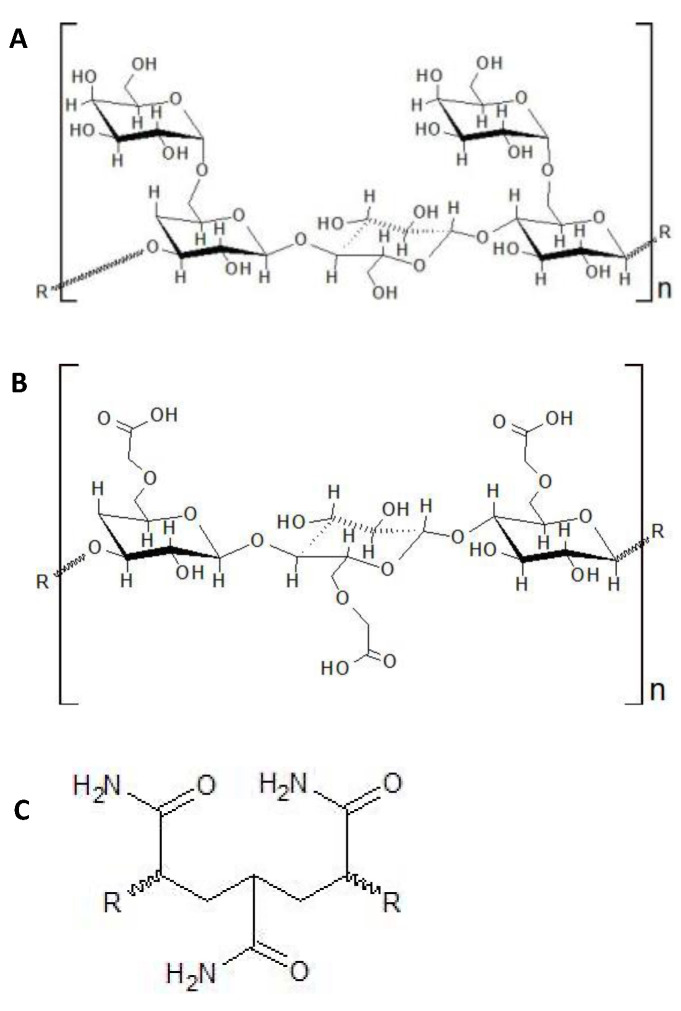
Chemical structure of (**A**) guar gum, (**B**) CMC, and (**C**) PAM.

As hydraulic fracturing is a commonly used method for recovering hydrocarbons from unconventional reservoirs, it is imperative to consider ways of minimizing the negative effect of this industry on the environment, for example through the use of more ecologically friendly additives in fracturing fluids. With enzyme-based breakers receiving more attention of late for their use in the energy recovery industry due to their environmentally friendly nature compared to harsh chemical breakers, a body of literature is emerging describing the enzymes and mechanisms involved in the biodegradation of polymers commonly used in hydraulic fracturing processes. As such, this review summarizes the current state of knowledge about the biodegradation of the most commonly used polymers in hydraulic fracturing industry including guar gum, carboxymethyl cellulose (CMC), and PAM (including hydrolyzed PAM, or HPAM) (Figure 1). We first overview the experimental approaches (Section 2) that have been used to study the biodegradation of these polymers so that the reader can readily follow the summary of experiments that have been done to better understand the biodegradation of these polymers and the efforts made to identify the key enzymes involved (Section 3). We then discuss the advantages and challenges of applying enzyme breaker technologies to oil and gas operations (Section 4), and sum up the review by articulating some major gaps in knowledge that can be addressed in future research (Section 5).

## 2. Approaches Used to Study Polymer Biodegradation

As polymers are high-molecular-weight substances, assessing their biodegradation requires the use of a variety of techniques that are somewhat unique to these large substrates. For example, the microbial utilization of polymers such as xanthan gum, guar gum, PAM, or CMC is generally not assessed using concentration measurements (typically used for lower-molecular-weight substrates) but rather are assessed by measuring changes in their physical properties including viscosity, molecular weight, total organic carbon (TOC), and/or polymer size, typically before and after a given incubation period. Viscosity and molecular weight reduction is frequently used as an indication that a polymer backbone has been broken down into smaller fragments [11,14,15]. For instance, the biodegradation of HPAM can be assessed using a rheometer to measure viscosity at a given temperature and shear rate [14,15]. The molecular weight and size of polymers such as PAM or CMC can be determined by gel filtration [16,17] or size exclusion [18,19] high performance liquid chromatography (HPLC). Changes in TOC are commonly assessed in polymer solutions by measuring the amount of carbon dioxide produced from the oxidation of the organic carbon, usually with an infrared gas analyzer [20]. Chemical methods can also be used to determine polymer degradation based on turbidity changes caused by the reaction of the polymer or one of its components with a specific reagent [21]. For example, xanthan gum can be detected by a colorimetric assay that measures the total amount of sugar units that are formed when the polymer is broken down with sulphuric acid; the carbohydrates then react with phenol to form a colored complex [22]. PAM biodegradation is commonly measured by the starch-cadmium iodine assay which is based on the conversion of amide (NH_2_) groups of a PAM molecule to *N*-bromoamides [23,24]. This method does not directly measure the cleavage of the PAM carbon skeleton, but it is commonly applied to assess the degree of polymer hydrolyzation.

**Figure 2:**
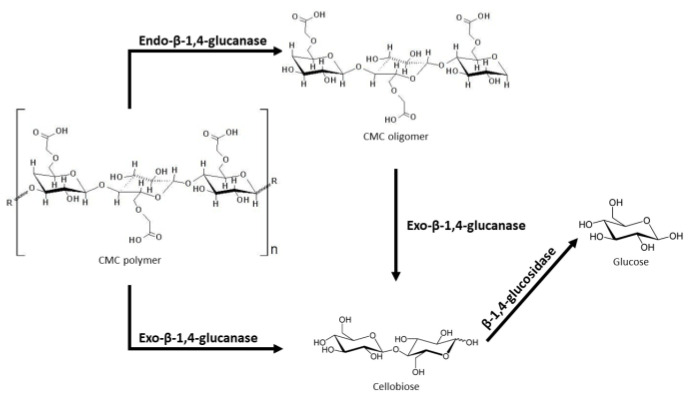
CMC biodegradation and enzymes involved in the attack of CMC linkages [31].

In addition to chemical approaches, the biodegradation of polymers can be determined by measuring the activity of the enzymes that directly catalyze the cleavage of a polymer chain. For example, cellulase activity can be assessed using the dinitrosalicylic acid assay, which measures the amount of reducing sugars (e.g., d-glucose) produced during CMC biodegradation. The measurements of reducing sugars from the hydrolysis of CMC is a method that was developed by Miller [25], wherein glucose will react with 3,5-dinitrosalicylic acid under alkaline solution to form 3-amino-5-nitrosalicylic acid, which produces a yellow color that strongly absorbs at 540 nm [26,27]. Although used for several decades, this technique has some limitations as it does not allow for the measurements of a specific enzyme, as only the end-product is measured. Therefore, it is impossible to identify the presence of a specific enzyme in a bacterial culture [26], and enzyme activity results tend to be overestimated [27]. Another enzyme assay relies on two different substrates, 4-nitrophenyl-β-d-cellobioside (pNPC) and 4-nitrophenyl-β-d-glucopyranoside (pNPG), to assess the enzyme activity of the three enzymes involved in biodegrading CMC (Figure 2). This simple assay is based on the hydrolysis of the bonds linking the sugar monomer (pNPG) or dimer (pNPC) with *p*-nitrophenol (gluconic bond), which exhibits a yellow color when in alkaline solution. pNPC can be hydrolyzed by both glucanase and the glucosidase (Figure 2); therefore, d-glucono-1,5-δ-lactone must be added in this assay, a specific inhibitor of the glucosidase [27,28]. With the addition of this substrate, pNPC is suitable for measuring the activity of both enzymes that direct attack the polymer, the exo-β-1,4-glucanase and the endo-β-1,4-glucanase [27,28]. pNPG is specifically hydrolyzed by the β-1,4-glucosidase. Therefore, this assay allows for the specific activity measurements of the glucanases and glucosidases involved in CMC degradation. Other analytical techniques that can be used to determine changes in the chemical structure and the functional groups of polymers include Fourier Transform Infrared Spectroscopy (FT-IR) [29] and Proton Nuclear Magnetic Resonance (1H-NMR) [20]. Metabolites formed from the microbial degradation of PAM have also been identified by HPLC and GC/MS (gas chromatography/mass spectrometry) analysis [17,29,30]. A good practice when assessing the biodegradation capabilities is the use of proper controls including abiotic controls (e.g., without addition of microbial inoculum) and polymer-free controls (e.g., incubations that contain the source of microorganisms but not the polymer substrate). The preparation of these controls in parallel with the tested microbial cultures will account for non-biological degradation and possible degradation of other carbon sources that could potentially be present in initial environmental samples commonly used to assess polymer biodegradation such as wastewater sludge, soil, and oilfield produced waters. The various methods overviewed in this section were used in many of the studies described below to determine the biodegradation of guar, CMC, and PAM/HPAM and identify associated enzymes of interest.

## 3. Polymer Biodegradation—Current State of Knowledge

Polymers including guar gum and related compounds, cellulose-based gels, and acrylamide-based polymers (PAM and HPAM) are used in the petroleum industry mainly for increasing viscosity and reducing friction in the reservoirs during water flooding and hydraulic fracturing. Though other polymers are also used (Table 1), in this review we focus on the description and development of enzymes involved in the biodegradation of guar gum, CMC, and PAM/HPAM (Table 2), as they are most frequently used polymers in oil and gas recovery operations. The sections below overview the main findings of many different studies that have aimed to describe the biodegradation of these polymers and/or identify the key enzymes that may be used for oilfield applications.

### 3.1. Guar Gum

Guar gum is typically extracted from the seed of the guar plant, *Cyamopsis tetragonolobus*, and it is widely used in many applications, particularly in the food industry [52]. Guar and its derivatives are also commonly used in hydraulic fracturing processes, although it has been reported that the polymer is sensitive to prolonged heat and as such does not keep its rheological properties [53]. Guar is a polysaccharide consisting of mannose units, bound together by β-1,4 glyosidic bonds and randomly attached galactose molecules linked to the mannose backbone by α-1,6 linkages (Figure 1A) [54]. The ratio of mannose compared to galactose units is usually between 1.8:1 and 2:1, and depends on the provenance of the polymer [52]. The rheological properties of guar gum depend on the length of the backbone and the mannose:galactose ratio [55]. The biodegradation of guar gum is catalyzed by hydrolases that attack the β-1,4 and α-1,6 linkages, by β-1,4-mannanase, β-mannosidase and α-1,6-galactosidase, resulting in its degradation into simple monosaccharides and disaccharides [37]. The β-1,4-mannanase cleaves the mannosidic bonds, having a direct effect on the viscosity of a guar solution as β-mannosidase act on the ends of the polymer chain and hydrolyzes the terminal glycoside group of guar gum. The α-galactosidase is responsible for the removal of the galactosidase units [55].

Guar-linkage specific enzymes (GLS) have been investigated for their use as enzyme breakers in hydraulic fracturing processes at different pH ranges, temperatures, and salinities. For example, GLS enzymes isolated from the fungus *Aspergillus niger* were shown to effective break (degrade) guar at temperatures between 15 to 60 °C, and at pH values of 3 to 11 [32] (Table 2). Thermostable α-1,6-galactosidase and β-1,4-mannanase enzymes were isolated from the hyperthermophile *Thermotoga neapolitana,* and were shown to be active at temperatures of up to 100 °C [37]. Similarly, *Thermotoga maritima* was reported for its expression of a thermostable α-galactosidase enzyme at 85 °C [36]. Another thermophilic bacterium isolated from hot springs, *Rhodothermus marimus*, showed galactomannan degradation activity by expressing a mannanase having an optimal activity at 85 °C and a pH of 5.4 [39]. A lower temperature (50 °C) mannanase isolated from *Enterobacter* sp. has also been reported to be an effective enzyme breaker for guar gum at a pH range of 3.0 to 8.0, and at high salt concentrations (up to 4 M NaCl) [38] (Table 2). Fridjonsson et al. [35] also reported an α-galactosidase from the genus *Thermus brockianus* ITI360 cloned into *E. coli* that was active at an optimal pH of 5.5 to 6.5 and optimal temperature of 93 °C. More recently, a protein-engineered galacto-mannanase was identified that was able to degrade the polymer up to 120 °C, which is the highest recorded temperature at which enzymes were active to degrade guar [34]. Similarly, the enzymes α-amylase and β-glucanase that are able to degrade xanthan gum and starch-based polymers, have also been found to be effective at temperatures up to 90 °C [41]. The long-term effectiveness of GLS enzymes to degrade guar-based filter cakes has been tested in oilfields relative to persulfate oxidizers, summarized by Brannon et al. [13]. To our knowledge, of the polymers we discuss in this review, only the GLS enzymes have been field-tested and found to be successful as filter cake breakers.

### 3.2. Cellulose-Based Polymers

Due to the fluctuation in price and occasional supply shortage of guar gum, other gelling agents are now often added to hydraulic fracturing solutions, including cellulose-based polymers such CMC and carboxymethylhydroxyethylcellulose (CMHEC) [56]. Azizov et al. [ 57] showed that the use of CMC in fracturing fluid systems can result in similar production performance and lower cost relative to guar gum. With increased industry interest in using polymers such as CMC in hydraulic fracturing operations, understanding the biodegradation of cellulose-based polymers is important for developing new enzyme breakers against these types of filter cakes.

Cellulose is a polymer that consists of repeating units of glucose linked by β-1,4 bonds [58] and CMC consists of a cellulose molecule with random carboxymethyl groups replacing hydroxyl groups within the molecule (Figure 1B) [59]. The chemical reaction for the replacement of the hydroxyl groups involves an alkali-catalyzed reaction with chloroacetic acid. The carboxymethyl groups render CMC soluble and chemically reactive [60]. The biodegradation of CMC occurs mostly via a cellulose-enzyme complex, known as a cellulosome, that includes: exo-β-1,4-glucanases (1), endo-β-1,4-glucanases (2) and β-1,4-glucosidases (3) [61] (Figure 2). Cellulosomes are complexes of cellulases bound to different scaffolding proteins such as carbohydrate-binding modules, docking modules, cohesion modules, and surface layer homology modules [62]. Exoglucanases attack the end of the CMC molecule, resulting in glucose or cellobiose formation, endoglucanases break down internal glucosidic bonds, and glucosidases catalyze the hydrolysis of cellobiose, forming glucose (Figure 2) [61,62]. Although many anaerobic bacteria express these multi-protein complexes, some anaerobes also hydrolyze cellulose or related molecules by expressing a single enzyme [63]. As the action of endoglucanases lowers the molecular weight of CMC, thus decreasing its viscosity, these enzymes are the ideal candidates for degrading the CMC-based filter cakes in oil reservoirs.

The production of endoglucanases from both fungi [64] and bacterial species [65] have found widespread application in the food and agricultural industries. In contrast, comparatively few studies have examined the development of endoglucanases for applications in the petroleum energy industry. Therefore, there is a limited understanding of the activity of endoglucanases under the environmental conditions that characterize subsurface petroliferous reservoirs, such as low redox conditions, and high salinities, temperatures, and pressures. While enzyme breakers have been developed to degrade cellulose-based polymers at temperatures between 15 to 60 °C and pH between 1 to 8 [56,57], most studies on the biodegradation of CMC filter cakes have been conducted with purified enzymes. However, their properties and identities are usually kept confidential (e.g., reported in patents) [66,67,68], thus limiting the progress in CMC enzyme breaker technology development. Trabelsi et al. [51] observed a viscosity decrease in guar and CMC when two different enzymes were tested as breakers at low pH (4.75), and at a relatively high temperature (49 °C). However, the protein sequences of the enzymes, nor their microbial origins, were reported [51]. Recently, CMC-degrading enzymes were retrieved from a thermophilic (50 °C), methanogenic enrichment culture established from cattle manure that was supplemented with CMC as its sole carbon and energy source. Extracellular enzymes degrading CMC were able to completely hydrolyze the polymer under high temperatures (50 to 80 °C), high salinities (up to 20% (*w*/*v*) salts), and were active between pH 5 to 8 [31]. Additionally, these enzymes could reduce CMC viscosity under high pressures (up to 4000 psi). The CMC-degrading enzymes from this anaerobic culture were subsequently isolated and purified for further study and testing [50]. These latter two studies showed that CMC-degrading enzymes can potentially be used as filter cake breakers under realistic oil field conditions characterized by high salinities and temperatures, though scale up and field tests are still required.

Recent advances in proteomics have helped understanding the structure and function of cellulosome complexes which can be used for cellulose biodegradation by some anaerobic bacteria [62,63]. New techniques to isolate proteins involved in the cellulosome complex have been developed from studying the structure of the complex. Work done by Hong et al. [69] reported a new technique to isolate and purify cellulases based on the affinity of the carbohydrate-binding module to amorphous cellulose. Han et al. [70] also recently surveyed different genetic modifications such as directed evolution or chemical modifications that can be done on thermo-stable enzymes to increase their efficacy at degrading their substrates in conditions that are considered more extreme. One example highlighted in this review is work done by Goldsmith and Tawfik [71], who used direct evolution via random mutagenesis to increase the stability of an endoglucanase from *T. reesei* QM 9414. After engineering the enzyme, its thermal stability increased such that the enzyme was active at 55 °C for 30 min and was more stable at a wider pH range (4.4 to 8.8). The interest in using CMC as an alternative to guar gum as a gelling agent in the past years [51,57] and the recent advances in proteomics and genetics offer a great opportunity to increase the research on this topic such that cellulose-based enzyme breakers can be reliably applied in oil recovery field operations.

### 3.3. PAM and HPAM

**Figure 3:**
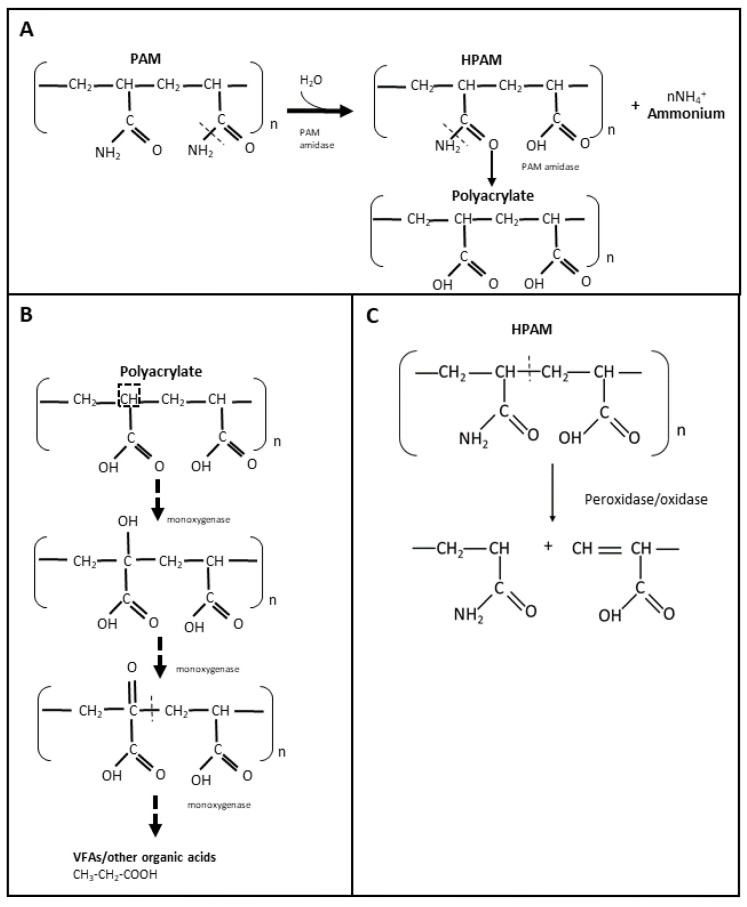
Microbial utilization of PAM and HPAM through hydrolyzation with amidase (**A**), suggested biodegradation of PAM by oxidation with monooxygenases (**B**), and PAM degradation mechanism by radical-forming enzymes (**C**).

The use of non-hydrolyzed and hydrolyzed polyacrylamide (PAM and HPAM, respectively) in hydraulic fracturing fluids has increased within the last 5 years, especially in North America [72]. Therefore, it is important to understand the biodegradation of PAM and HPAM in order to develop potential enzymes that can be used as breakers to treat these types of polymer filter cakes. PAM is a high molecular weight polymer that is synthesized by polymerization of acrylamide, either as a linear chain or as a crosslinked structure [21] (Figure 1C). Due to its high molecular weight and stable carbon backbone, PAM has been considered relatively resistant to microbial biodegradation [45,73]. It is believed that PAM and HPAM are unable to pass through microbial cell membranes, and that their carbon skeleton is difficult to access by microorganisms [74]. Nevertheless, the microbial utilization of PAM and HPAM has been reported since the late 1990s, including by microorganisms from soil, activated sludge, and oilfield production/injection waters. The amide (-NH_2_) groups of PAM and HPAM can be hydrolyzed and converted into ammonium (NH_4_^+^) (Figure 3A), which can then be used as a source of nitrogen for microbial growth [46,47,74]. Both aerobic and anaerobic microorganisms have been shown to utilize PAM and HPAM as nitrogen sources [29,75,76,77,78,79]. PAM or HPAM hydrolysis is catalyzed by a specific amidase enzyme, which has been repeatedly detected in microbial cultures amended with these polymers [19,74,76,79] (Figure 3A). In addition, chemical analyses have shown that during microbial utilization of PAM, its amide groups can be converted into a carboxylic acid (COOH) [17,29,30,77,79], resulting in the formation of polyacrylate. However, the utilization of the NH_2_ groups from PAM/HPAM (deamination) does not lead to a decrease in the viscosity or molecular weight of the polymers as the carbon-carbon backbone is not cleaved [73], and therefore amidases are not good targets for developing enzyme breakers for PAM and HPAM.

The microbial utilization of PAM or HPAM as a carbon source is considered more challenging. Only the partial biodegradation of PAM or HPAM has been reported, although the lack of polymer-free controls in some of these studies does not unequivocally confirm if biodegradation was occurring. According to Nakamiya and Kinoshita [20], soil and activated sludge isolates degraded up to 20% of PAM after 27 h of incubation. Wen et al. [78] measured a 70% PAM removal efficiency by two *Bacillus* isolates after 96 h of incubation, but this degradation efficiency was assessed based on the starch-cadmium iodine assay, which measures the removal of amide groups from PAM, rather than carbon-carbon bond cleavage. Similarly, Bao et al. [29] obtained bacterial cultures from oilfield produced waters with a HPAM removal efficiency of 14%, but the cleavage of the carbon backbone in HPAM was not directly shown. More recently, microbial communities from a combined aerobic and anaerobic reactor system were shown to decrease HPAM viscosity by up to 78% [15]. In addition, the authors observed two compounds with lower molecular weight than HPAM in the aerobic system using GPC spectra [15]. Other recent studies [72,77,80,81,82] reported the presence of volatile fatty acids such as propionate, acetate and formate in microbial cultures utilizing HPAM under anaerobic conditions. Thus, it is believed these fatty acids accumulate as a result of HPAM biodegradation [72,77].

Despite the number of studies reporting PAM or HPAM biodegradation, only a few have identified specific enzymes potentially involved in the breaking of the carbon skeleton, but they should be carefully examined. Dehydrogenase and oxidases were detected in microbial systems designed for the treatment of HPAM [80,81]; however, it cannot be discerned whether these enzymes were solely produced for the purposed of HPAM biodegradation since additional carbon sources (such as glucose) were present in the systems. More recently, Song et al. [83] measured the activity of laccase (oxidase) and dehydrogenase in a combined aerobic and anaerobic bioreactor that was initially amended with glucose and urea, and then conditioned with HPAM. In this study, authors observed that laccase activity was independent of the HPAM concentration and dehydrogenase activity was indirectly proportional to the concentration of HPAM [83]. From several reports on PAM/HPAM biodegradation, it is hypothesized that the biodegradation of these polymers occurs by initial oxidation reactions that would first add a hydroxy (-OH) group into the alpha carbon of HPAM and a ketone (=O) group to allow the subsequent cleavage of the PAM/HPAM carbon skeleton, through the activity of oxygenase enzymes (e.g., monooxygenases) (Figure 3B). However, further studies are required to confirm whether these enzymes are indeed present in PAM-biodegrading cultures and if they can be used as enzyme breakers to degrade HPAM or PAM polymers in oilfield systems. A recent study added to the skepticism that HPAM or PAM polymers can be used as a carbon source [84]. Repeated transfers of microbial communities enriched from activated sludge and oilfield produced water and incubated under thermophilic conditions did not reduce the polymers’ viscosity when PAM or HPAM were provided as sole carbon sources. Instead, these polymers were shown to serve as nitrogen sources when an alternate carbon source such as glucose was provided [84].

PAM or HPAM degradation has also been reported when commercial or extracted enzymes were directly added to polymer solutions. Gupta [48] patented an enzyme breaker known as asparaginase to degrade PAM. The authors reported that this enzyme was able to deaminate the amide group of PAM and subsequently cleave the polymer, resulting in a viscosity decrease. However, the specific mechanism involved in ‘breaking’ the carbon skeleton of PAM was not shown. Other extracellular enzymes such as oxidases or peroxidases may also be effective as PAM/HPAM breakers, wherein free radicals are formed that react with the polymer carbon, leading to a cleavage in the carbon skeleton of the polymer (Figure 3C). Initially, Ramsden et al. [44] observed the degradation of a 0.5% PAM solution at 20 °C when a commercial xanthine oxidase was added in the presence of xanthine. Nakamiya et al. [43] subsequently reported the degradation of PAM when using a purified hydroquinone peroxidase enzyme isolated from *Azotobacter beijerinckii* HM121. In the presence of tetramethyl hydroquinone and hydrogen peroxide, this peroxidase was able to degrade PAM into polymers of smaller molecular weight within an hour of incubation at 30 °C [43]. Hydroquinone peroxidase was believed to react with hydrogen peroxide to form hydroxyl radicals which then reacted with tetramethyl hydroquinone to form a corresponding radical that attacked the carbon chain of PAM and by hydrogen abstraction broke the polymer chain [43]. Recently, Gilbert et al. [42] observed that horseradish peroxidase (HRP), in the presence of hydrogen peroxide, can also catalyze the degradation of HPAM at 37 °C by free radical formation. After 24 h, HRP decreased the viscosity and molecular weight of the HPAM solution by 81% and 67%, respectively, in the presence of 97 mM peroxide [42]. Both molecular weight and viscosity reduction were dependent on the concentration of hydrogen peroxide [42]. The results of the above studies suggest that these free radical-forming oxidases and peroxidases could potentially be used for degrading PAM or HPAM polymers in oil reservoirs, at least at mesophilic temperatures between 20 to 37 °C. However, possible interactions between the radical components formed from the potential enzymes and other chemicals present in the reservoir are yet to be investigated to confirm the effectiveness of these types of enzymes for application in oil and gas fields.

## 4. Advantages and Challenges of Applying Enzyme Biotechnologies to Oil and Gas Recovery Operations

The use of enzyme breakers for polymer degradation has many advantages relative to chemical breakers. Enzymes are biologically produced, they are generally non-toxic and overall are considered more environmentally friendly [85], contrary to many chemical breakers, which can be toxic and damaging to reservoir formations. Insoluble products and unreactive polymer chains can form from the use of reactive acids and persulfates and reactions are non-specific [86]. In contrast, enzyme breakers bind specific polymers, thus are less likely to form unreactive strands that can cause formation damage [10]. Enzymes have a longer reaction time than chemical breakers, since they can regenerate and will continue to catalyze a reaction, as long as they are not denatured and their active site is still available [85]. The ability of the enzymes to regenerate also implies that smaller amounts of enzyme breaker can be added to fracturing fluids relative to chemical breakers [11]. As such, Kyaw et al. [87] observed lower residue formation and more homogeneous degradation of a guar-based polymer when an enzymatic breaker was used relative to a commercially available chemical oxidizer, resulting in a lower concentration of enzyme required.

One of the challenges of using enzymes for polymer degradation in hydraulic fracturing operations is that they must withstand the extreme conditions present within oil reservoirs. Enzymes can be more sensitive to extreme temperatures, pressures, salt concentrations and high pH conditions compared to chemical oxidizers. However, laboratory and field studies over the past two decades have demonstrated that these challenges can be overcome with better isolation strategies, and new advances in proteomics and molecular biology. Although some enzymes denature above mesophilic conditions (e.g., at greater than 45–50 °C), enzymes isolated from thermophilic organisms have shown continued polymer degradation at higher temperatures. Successful long-term reservoir treatment using GLS enzymes at high temperature (up to 150 °C) was reported in various hydraulically fractured reservoirs across the United States [13]. Moreover, numerous studies have now shown that enzymatic activity can occur under a variety of salinities, and pH ranges. For example, Cobianco et al. [41] reported that amylase and glucanase enzymes tested for their ability to degrade starch and xanthan gum polymers, were still active when using various brines (e.g., KCl, CaCl_2_, HCOONa) and salinities ranges (3%, 6%, and 10%).

Advances in genetics and proteomics are also allowing for enzyme optimization, for example by improving enzyme production or gene enhancement to resist specific reservoir conditions. For example, Armstrong et al. [11] optimized a polymer-specific enzyme for commercial production, increasing enzyme yield and the ability to withstand high temperatures with genetic enhancement. Insights from studies on thermostable endoglucanases designed for other biotechnology applications such as biofuel production (as reviewed by Yennamalli et al. [88]) could also help to improve the development of enzyme breakers for cellulose-based polymers in the fossil energy sector.

Another challenge in the use of enzyme breakers is the ability to control the break time and maintain the stability of the enzyme until it needs to react with the polymer [40]. Ideally, enzyme breakers are added to fracturing fluids after proppant delivery and fracturing formation has occurred [32]. Recently, a variety of strategies have been developed to overcome reactions times and stability issues in enzyme breakers. Chopade et al. [40] reported that the addition of lignosulfonates helped to stabilize the activity of the guar-degrading enzymes at temperatures up to 70 °C and pH values up to 10.5. Moreover, Barati et al. [89] reported a protective nanoparticle system developed to maintain the stability of the enzyme breakers at alkaline conditions and resist elevated temperatures, while delaying the reaction time of the breakers. Other strategies suggested to maintain enzyme stability and control reaction time include encapsulation methods that use polyvinylidene chloride or nylon materials [14]. Overall, new advances in biological sciences, biochemistry, and engineering can be applied to overcome the challenges related to enzyme stability under reservoir conditions and enzyme delivery.

## 5. Summary and Gaps in Knowledge

Enzyme biotechnologies, specifically the use of enzyme breakers, have great potential for use in oil and gas applications because they are biologically produced and offer a more environmentally friendly option for in situ polymer degradation relative to chemical oxidizers. With the increase in hydraulic fracturing activities in the past decade, especially in North America, there is a great need for the ongoing development of enzyme breaker solutions that can degrade a variety of polymers now applied in hydraulic fracturing fluids. Endoglucanases able to degrade cellulose-based polymers such as CMC have been widely developed for use in the food and agriculture sectors, but fewer endoglucanases have been commercially produced for application in the oil and gas sector. Recent studies have shown that radical forming enzymes such as oxidases and peroxidases, well studied for other industrial applications, can degrade PAM solutions at mesophilic temperatures. Thus, oxidases and peroxidases could be targeted for development as potential enzyme breakers to degrade PAM-based polymers in higher temperature systems. Many of the studies summarized in this review have been conducted in a laboratory setting under ambient pressures and temperatures and as such show great promise for the use of enzymes to degrade polymers. However, comparatively little is known regarding their ability to function as breakers under the higher temperature, pressure, and salinities usually encountered in oil and gas reservoirs, representing a major gap in knowledge to address in future research.

Enzyme technology development encounters many challenges during the isolation, optimization, and production processes. Understanding the mechanism(s) that initiate the attack of a polymer structure such that its viscosity and molecular weight decrease is key to developing a successful enzyme breaker. Advancements in genetics and biochemistry (e.g., proteomics) used for enzyme optimization in other industries (such as for biofuels) can also be applied to the oil and gas sector. Enzymes can be optimized to improve their stability at specific reservoir conditions such as low pH and high salinity ranges, and enzyme yields can be increased to a commercial scale by enhancing specific genes coding the targeted enzymes, thus helping overcome economic challenges related to production. As summarized in this review, though many studies have described the development of enzyme-based polymer breakers that are promising, applied research focused on understanding enzyme regulation and the mechanisms of enzyme optimization for currently used polymers is still required to accelerate enzyme breaker technology development in the oil and gas sector, particularly under the most appropriate environmental conditions (e.g., high temperatures, salinities, and pressures). Finally, conducting field trials with any developed enzyme breaker is critical to evaluating their field use. While guar-based enzyme breakers have been field-tested with some success [13], similar field trials are still required to test enzymes that degrade cellulose-based and acrylamide-based filter cakes. Future research that addresses these knowledge gaps will ultimately allow for the replacement of toxic and hazardous products such as oxidizers with more ecologically friendly enzymes in the oil and gas recovery industry.

## Figures and Tables

**Table 1:** Most commonly used polymers and their use in different steps of the hydraulic fracturing process.

Type of Polymer	Gelling Agent	Use	References
**Cellulose-based**		Proppant delivery agent	[8]
	Hydroxyethyl cellulose	Fluid loss additive	
		Gravel packing	
		Thickener	
	Hydroxypropyl cellulose	Proppant delivery agent	[8]
		Thickener	
	Carboxymethyl cellulose	Proppant delivery agent	[8]
		Gravel packing	
		Thickener	
	Carboxymethylhydroxyethyl cellulose	Proppant delivery agent	[8]
	Methyl cellulose	Thickener	[8]
**Guar-based**	Guar gum	Proppant delivery agent	[8]
		Thickener	
	Hydropropyl guar	Proppant delivery agent	[8]
		Gravel packing	
	Carboxymethyl guar	Proppant delivery agent	[8]
	Carboxymethylhydropropyl guar	Proppant delivery agent	[8]
**Acrylamide & acrylic acid-based**	Polyacrylamide	Friction reducer	[7,8]
		Thickener	
	Polyacrylate	Friction reducer	[8]
	Methylacrylamide	Thickener	[8]
	Acrylic acid	Thickener	[8]
	Methylacrylic acid	Thickener	[8]
	Copolymers from acrylamide & acrylic acid	Friction reducer	[8]
		Thickener	
**Others**	Xanthan gum	Proppant delivery agent	[4,8]
		Foaming agent	
		Gravel packing	
		Drilling muds	
		Thickener	
	Starch & its derivatives	Fluid-loss additive	[4]
	Scleroglucan	Proppant delivery agent	[8]
	Polyurethanes, Polyesters,	Thickeners	[8]
	Locust bean gum, Gum Ghatti		
	Gum karaya, Tragacanth gum		
	Tamarind gum		
	Welan gum	Foaming agent	[8]
		Thickener	
	Polycationic quaternary amine polymer,	Clay stabilizers	[8]
	Guanidyl copolymer, Anionic polymer,		
	Copolymer of styrene & maleic anhydride		
	with polyethylene glycol		
	Lignosulfonate	Fluid-loss additive	[8]
	2-Acrylamino-2-methy-1-propane sulfonic acid (AMPS) derivatives & N-Vinylpyridine	Thickeners	[8]

**Table 2:** Enzymes that degrade guar, xanthan gum, and CMC polymers used in hydraulic fracturing applications, and potential enzymes for degrading acrylamide-based polymers.

Polymer	Enzyme Name	Activity Conditions (Temperature, pH, Salinity)	Source of Enzyme	Reference
**Guar gum**	1,6-α-d-galactosidase	10 to 82 °C, pH 2 to 11	*Aspergillus niger*	[32]
	Mannan endo-1,4-mannosidase			
	Mannanase II	40 to 70 °C, pH 7 to 8.5	Not specified	[33]
	Galacto-mannanase	up to 120 °C	Not specified, but gene expressed in *E. coli*	[34]
	α-1,6-galactosidase	93 °C, pH 5.5 to 6.5	*Thermus brockianus*	[35]
	α-1,6-galactosidase	85 °C	*Thermotoga maritima*	[36]
	α-1,6-galactosidase	85 to 100 °C pH 7.4	*Thermotoga neapolitana*	[37]
	β-1,4-mannanase			
	Mannanase	50 °C, pH 3 to 8, up to 4 M NaCl	*Enterobacter* sp. N18	[38]
	Mannanase	85 °C, pH 5.4	*Rhodothermus marimus*	[39]
	Mannanase	60 to 70 °C, pH up to 10.5	Not specified	[40]
**Starch**	α-amylase	50 to 90 °C, pH 5–9	Not specified	[41]
**Xanthan gum**	β-glucanase			
**PAM/HPAM**	Horseradish peroxidase	37 °C	*Amoracia rusticana*	[42]
	Hydroquinone peroxidase	30 °C, pH 7	*Azotobacter beijerinckii* HM121	[43]
	Xanthine oxidase	20 °C	Bovine milk	[44]
	Phosphatase, Urease, Dehydrogenase	33 °C, pH 7.5	Activated sludge *	[45]
	Amidase	38 °C, pH 6.6	*Klebsiella* sp.	[46]
	Urease	24 °C, pH 8.19	*Bacillus megaterium*	[47]
	Asparaginase	20 to 120 °C	*Aspergillus oryzae*	[48]
	Laccase	35 °C	Wastewater enrichment *	[36]
**CMC**	Endo(1,4)-glucanase-d-xylanase	15 to 60 °C, pH 1 to 8	Not specified	[49]
	Exo(1,4)-glucanase-d-xylanase			
	Xylanase	50 to 80 °C, pH 6 to 8, up to 20% (w/v) NaCl	*Caldicoprobacter faecalis*	[31,50]
	Enzyme 1 and enzyme 2	49 °C, pH 4.75	Not specified	[51]

* Enzyme was detected but not isolated.

## Data Availability

Not applicable.
